# Body Mass Index Trends among a Cohort of Subjects Enrolled in Medication-Assisted Treatment Programmes for Opioid Use Disorder: Racial/Ethnic, Gender, and Age Differences

**DOI:** 10.31487/j.JFNM.2022.01.01

**Published:** 2023-05-24

**Authors:** Juhi Saxena, Rachana Chilakapati, Peter Attia, Daniel M. Rosenblum, Stanley H. Weiss

**Affiliations:** 1 Department of Medicine, Rutgers New Jersey Medical School, Newark, New Jersey, USA; 2 Department of Biostatistics and Epidemiology, Rutgers School of Public Health, New Jersey, USA

**Keywords:** Opioid use disorder, obesity, overweight, underweight, BMI, methadone, medication-assisted treatment, programmes, COVID-19, SARS-CoV-2, diabetes mellitus, New Jersey Behavioral Risk Factor, Survey, National Health Interview Survey, cohort study, cannabis use, alcohol use

## Abstract

**Introduction:**

Opioid use disorder (OUD) and obesity are two pressing public health concerns in the United States (US). However, the relationship between these two epidemics has not been well-studied. Our study aims to describe the prevalence rates of obesity in individuals with OUD from a cohort study and compare that to the expected prevalence that would be observed based upon New Jersey state and US population survey data. Additionally, we sought to study whether Body Mass Index (BMI) distribution in this cohort varied by race/ethnicity, gender, and age.

**Methods:**

Our subjects (N=151) are part of a drug user cohort study of persons enrolled in medication-assisted treatment (MAT) programmes in New Jersey. Using the New Jersey Behavioral Risk Factor Survey (NJBRFS) and the National Health Interview Survey (NHIS), we generated expected BMI distributions based on race/ethnicity, age, and sex. Expected rates were compared to observed BMI. Standardized prevalence ratios were calculated, and 95% confidence intervals were constructed.

**Results:**

Among females, obesity was more prevalent in those with OUD than in the general US population. Among persons ≤50 years old, overweight and obesity were more prevalent in those with OUD than in NJBRFS. Persons who did not inject drugs were more likely to be overweight. The prevalence of underweight was significantly higher among Black non-Hispanic minorities, males, older subjects (aged 66-85), and persons who inject drugs.

**Conclusion:**

In our study, the trends in BMI vary based on race/ethnicity, gender and age in these patients with OUD. These varying trends highlight the need for tailored screening and prevention strategies. Primary care providers should be aware that their patients with OUD have multiple health problems that need to be addressed beyond their OUD condition itself. Providers are in a pivotal role to screen and implement interventions to improve their health outcomes.

## Introduction

Opioid use disorder (OUD) and obesity are two leading epidemics of significant public health concern in the United States. Americans alone consume 99% of the world’s hydrocodone supply and 80% of the world’s opioids supply [1]. Likewise, the obesity in adults has increased from 30.5% in 1999-2000 to 42.2% in 2017-2018 [2]. The intersection of these epidemics such as the prevalence of obesity in adults with OUD has been inadequately studied in the United States. Although, government surveys such as the National Health Interview Survey (NHIS) and the New Jersey Behavioral Risk Factor Surveillance System (NJBRFS) strive to capture data representing the overall health of Americans at the federal and state level respectively, individuals with OUD remain an understudied population. The increasing association of health consequences such as HIV, COVID-related health complications, and homelessness further merit public health efforts towards surveillance and prevention in this high-risk vulnerable population.

Many adults with OUD are treated in Medication-Assisted Treatment (MAT) programmes or by physicians in various modalities. MAT programmes primarily focus on drug addiction prevention and recovery with the use of methadone, a μ-opioid receptor agonist, in combination with behavioural therapies [3]. The connection between methadone treatment and weight gain is greatly debated in current scientific literature. Methadone treatment has been associated with weight gain as well as metabolic and endocrine changes, including changes in glucose metabolism [4-6]. When methadone activates μ-opioid receptors, it may increase one’s preference for sweet-tasting foods [7]. In one nutritional study, 90% of patients detoxing from heroin craved sweets; in another study, 60% reported a sugar craving while on methadone [8, 9]. Preference for foods with high sugar content may result in systematic weight gain. Neurochemical and brain imaging studies provide evidence that food addiction is similar to psychoactive drug addiction [10].

Drug addiction and food addiction share underlying biological mechanisms related to how the brain responds to reward compulsive consumption behaviours [11, 12]. Addictive drugs increase dopamine released in the striatum and comparative dopaminergic responses may play a role in the rewarding effects of food consumption. This dopaminergic reward system may contribute to excessive consumption of food and subsequent obesity [13]. Furthermore, a correlation between long-term methadone treatment and metabolic syndrome has been observed [14]. BMI increase over time has also been observed independent of methadone blood levels and dosages [15]. Unfortunately, treatment for associated chronic illnesses like obesity often do not receive sufficient medical care at these treatment programmes. This selective treatment may negatively impact patient’s overall recovery and well-being.

OUD-associated obesity needs to be studied further in connection to coronavirus disease 2019 (COVID-19) because concurrent obesity and COVID-19 have been consistently associated with adverse health outcomes [16]. Obesity affects innate immunity mechanisms and increases risk for development of infection, which might explain why patients with obesity are more prone to suffer from respiratory infections in the context of COVID-19 [17]. Individuals with OUD also often encounter complications due to underlying health conditions, such as obesity, cardiovascular disease, lung disease, and a compromised immune system, and all these conditions are risk factors for COVID-19 infection [18-21]. The intersectionality of race, socioeconomic status, and gender, along with poor health status due to comorbidities, has led to increased mortality during the COVID-19 pandemic. COVID-19 has disproportionately impacted racial and ethnic minorities, with particularly higher rates among African Americans [22]. Racial disparities leading to poor health outcomes existed prior to the COVID-19 pandemic, but the higher rates of COVID-19 among African Americans underscores this disparity. Racial inequalities reflected by socioeconomic status may be a predictor of COVID-19 to some degree [23].

African Americans make up a large part of essential workers, and individuals who are not able to work remotely and use public transportation to commute [23, 24]. These factors may contribute to a greater risk for contracting the virus. Understanding COVID-19’s impact on race/ethnicity is important for ensuring that vulnerable populations have access to COVID testing and vaccines. The high prevalence of obesity in patients with OUD and the adverse outcomes from COVID-19 in obese patients underscore the importance of providing support for the treatment and recovery of individuals with OUD as part of the strategy to control the COVID pandemic.

Studying the prevalence of obesity in drug users enrolled in MAT programmes is clinically relevant, and this information bears a significant impact on future healthcare interventions. This paper aims to investigate the prevalence rates of obesity in a drug user cohort as compared to NRBRFS and NHIS data. We compare obesity prevalence in patients with OUD directly to people in the general population matched by race/ethnicity, gender, and age. We hypothesized that we would project a higher prevalence of obesity in the OUD cohort and varying trends based on racial/ethnic, gender, and age groups.

## Methods

### I Drug User Cohort

From 2016 through 2018, we surveyed 298 patients enrolled at four medication-assisted treatment (MAT) centers in northern and central New Jersey. Patients were surveyed on their lifetime health experiences, as part of a baseline interview administered by trained personnel. After obtaining signed consent, patients’ treatment records were obtained from their specific clinic. The questionnaire used to interview patients was approved by the Rutgers Newark Health Sciences IRB. From these 298 completed interviews, we were unable to obtain medical records for 105 subjects, likely due to their leaving treatment facilities in the interim, resulting in their medical records being transferred or removed for confidentiality reasons. Of the 193 subjects for whom we recovered medical records, we were able to extract weight and height data to calculate BMI calculations for 151 subjects. Therefore, our paper’s analyses were based on 151 patients enrolled at the MAT programmes, which is 50.7% of our interviewed group. We calculated BMI using US metrics: BMI = [weight (lb) ÷ height^2^(in.)] × 703. We used the 1998 National Heart, Lung, and Blood Institute (NHLBI) terminology to classify BMI in the following categories of underweight (BMI of < 18.5), normal weight (BMI of 18.5-< 25), overweight (BMI of 25-< 30), and obese (BMI of ≥ 30).

Within our cohort of 151 patients, we created 7 age groups of roughly equal size between which BMI tended to change: 21-35, 36-40, 41-45, 46-50, 51-55, 56-60, 61-65, 66-85. Sex was binarily reported as male or female, as there were no intersex or transgender individuals in our cohort. Our study reports five racial/ethnic categories: White, non-Hispanic; Black, non-Hispanic; Asian, non-Hispanic; Hispanic/Latino; and Other, non-Hispanic. With this demographic information, individualized risks were calculated for each subject’s race/ethnicity, gender, age, and BMI within our cohort. Individual risks were calculated by totaling the individualized risks over specific categories, such as female or PWID [25].

### II Obesity Prevalence Data Collection

NJBRFS is the New Jersey (NJ) state-administered version of the CDC’s Behavioral Risk Factor Surveillance System (BRFSS) Questionnaire [26]. Using the NJBRFS’s embedded online data analysis tool, New Jersey State Health Assessment Data system, we extracted BMI classification distributions among adults aged 21-85 in NJ stratified by race, ethnicity, sex, and age group. BMI was classified using NHLBI terminology.

NHIS is a face-to-face survey designed to represent the civilian, non-institutionalized population of the US [27]. Using the same race/ethnicity/sex/age categorizations as the NJBRFS extraction, we categorized NHIS demographic and BMI data. BMI was classified using NHLBI terminology. We extracted survey data from the years 2011 to 2017, inclusive, from both NJBRFS and NHIS. All NHIS, NJBRFS, and extracted survey data were categorized using SAS^®^ Software, Version 9.4 (SAS Institute, Inc., Cary, NC).

### III Statistical Analyses

The extracted BMI category proportions, stratified by race/ethnicity, gender, and age group, from NJBRFS and NHIS, were imported into Excel spreadsheet to calculate the expected prevalence rates. These proportions were compared against the observed BMI and demographics of the 151 New Jersey MAT patients. We calculated Standardized Prevalence Ratios (SPRs) by dividing the observed BMI prevalence by expected BMI prevalence calculated from NJBRFS and NHIS. A SPR of 1.0 indicates the observed BMI proportions equals the expected BMI proportions. A SPR > 1.0 indicates higher observed BMI proportions and a SPR < 1.0 indicates higher expected proportions. The observed BMI was assumed to have a Poisson distribution and 95% confidence intervals for the SPRs were calculated [28]. We then used OpenEpi to calculate p-values for the SPRS [29]. All p-values in this paper, tables, and supplementary tables report the Fisher Exact two-tailed p-value.

### IV Further Analysis: Alcohol and Regular Cannabis Use

After performing the statistical analyses aforementioned, we identified alcohol and regular cannabis use as unexpected trends that may be factors affecting BMI. We categorized each individual’s historical alcohol use into three categories based on NHIS definitions – abstained, light/moderate, and heavy. The definition for heavy drinking was above 14 drinks a week for men or 7 for women, and for light/moderate drinking was any nonzero number below these cutoffs [27]. We calculated risk ratios (RRs) for BMI categories with alcohol use levels (N=151). The RRs compared the classification of underweight, overweight, and obese to the reference group normal weight, with the heavy drinking as an exposed group and the abstained category as a control. Similarly, regular cannabis use was analysed in these 151 subjects. Individuals self-reported no regular cannabis use or regular cannabis in the questionnaire survey. RRs compared the BMI classifications of underweight, overweight, and obese to the reference group normal weight. The RRs used regular cannabis as an exposed group and never regular cannabis a control. Table 6 further details the RRs for regular cannabis use and alcohol use with two-tailed p-values and Taylor series-based calculations of 95% CI.

## Results

From 2016 to 2018, BMI information was collected for 151 subjects in our drug user cohort. The cohort was 58.9% female, 39.1% Black non-Hispanic, and 42.4% obese. There were an equal number of cases classified as normal weight (26.5%) and overweight (26.5%), and the least number of cases in the underweight (4.6%) category. More than half of the cohort (54.3%) were persons who injected drugs (PWID). Most subjects attained an education level of some high school (31.1%) or graduated high school (26.5%) (see Table 1).

Mean methadone doses were reported for the N=146 subjects (see Table 1) and the 5 subjects not included in this analysis reported methadone was not a part of their treatment. The highest mean methadone dose was in Hispanic (90.5 mg/day, *N*=34) and other non-Hispanic subjects (94.0 mg/day, N=2) and the only Asian subject had the lowest dose (5.0 mg/day). Compared to the rest of the age ranges, 41-45 had the highest dose (98.3 mg/day) and 51-55 had the lowest dose (73.1 mg/day). In a two-sample independent t-test with pooled variance and normal weight as a control, the two-tailed p=0.09 [29, 30].

Our study compared the observed BMI prevalence rates of the drug user cohort (N=151) to the expected prevalence rates calculated from NJBRFS and NHIS survey data (see Table 2). Overall, underweight was significantly more prevalent (NJBRFS: SPR=3.52, p=0.009 and NHIS: SPR=3.98, p=0.005), overweight was significantly less prevalent (NJBFRS: SPR=0.70, p=0.024), and obesity was significantly more prevalent (NJBRFS: SPR=1.30, p=0.049) among the drug users than in the population. Black non-Hispanics in the cohort were significantly more likely to be underweight compared to the NJBRFS data (SPR=6.71, p=0.007) and NHIS data (SPR=7.36, p=0.005). Males in the cohort were significantly more likely to be underweight (NJBRFS: SPR=7.44, p=0.016 and NHIS: SPR=7.69, p=0.015). Females were significantly more likely to be obese compared to the NJBRFS data (SPR=1.44, p=0.026).

Subjects aged 66-85 had a higher underweight prevalence compared to NHIS data (SPR=13.91, p=0.019). Subjects >50 were significantly more likely to be underweight (NJBRFS: SPR=4.03, p=0.037 and NHIS: SPR=4.89, p=0.020). In comparison, subjects ≤ 50 were more likely to be overweight (SPR=0.61, p=0.043) and obese (SPR=1.68, p=0.005) compared to NJBRFS data. PWID had a significant higher underweight prevalence (NJBRFS: SPR=4.15, p=0.016 and NHIS: SPR=4.37, p=0.013) and non-PWID had a significantly higher overweight prevalence (NJBRFS: SPR=0.58, p=0.027). Tables 4 & 5 detail Obs/Exp ratios, two-tailed p-value and 95% CI.

The results of two risk ratio analyses comparing BMI categories for regular cannabis use and alcohol use respectively are presented in (Table 3). 51.7% of our cohort reported using cannabis regularly (see Table 3). Obese subjects were less likely to be regular cannabis users (RR=0.65, p=0.011, 95% CI: 0.47-0.90). Our cohort comprised heavy drinkers (44.4%), light/moderate drinkers (27.8%), and abstainers (27.8%). Obese subjects were less likely to be heavy drinkers (RR=0.63, p=0.030, 95% CI: 0.43-0.91).

## Discussion

Our study found significant differences in BMI prevalence in subjects enrolled in MAT programmes compared to state and national trends. We found a higher overall prevalence of overweight (26.5%) and obese (42.4%) individuals in our cohort compared to state surveillance data. Additionally, the overall underweight prevalence (4.6% of the study population) was significantly higher in our cohort compared to state and national data. These trends may provide insight for future physicians to assess high-risk factors for proper prevention and intervention.

We were surprised to find unexpected racial and ethnic trends that were strikingly different among these OUD than among state and national surveillance populations. National studies examining BMI show Black non-Hispanic adults have higher prevalence of overweight and obesity. In contrast, Black non-Hispanic cohort subjects were significantly more likely to be underweight than what was predicted by either NJBRFS (SPR=6.71, p=0.007) or NHIS data (SPR=7.36, p=0.005) [31, 32]. Additionally, half of the Hispanic subjects were obese. Hispanic subjects comprised a little over a fifth of the cohort population and although in our study the observed obesity prevalence in adult Hispanic subjects was not statistically significant, it was higher than reported in other studies done in the general adult Hispanic population of New Jersey (27.5%) and in the US (42.5%) [33].These findings highlight the need to screen and treat OUD as part of a strategy to manage the opioid and obesity epidemic. Age, race, and ethnicity should be used to stratify risk in individuals with OUD, especially considering their increased likelihood for more severe disease and mortality from contracting infections like COVID-19 and HIV.

Gender differences observed may be a result of different drug use history, age of initiation, duration, and type of drugs utilized. Our study found men tended to be more underweight than NJBRFS (SPR=7.44, p=0.016) or NHIS (SPR=7.69, p=0.015) rates, similar to a comparable study conducted among male drug addicts vs non-addicts in Dhaka, Bangladesh [34]. In contrast, females in our study had a higher prevalence of obesity than those calculated from NJBRFS data (SPR=1.44, p=0.026). This phenomenon could be due to the fact that older women are more likely to have a different drug use history than men, leading to a different set of health outcomes in the long term. For example, a 2014 study on the gender differences in self-reported BMI in drug users in Latin America found a positive BMI correlation with older women who were more likely to use over-the-counter analgesics and tranquilizers [35]. In the United States, which is a developed country, there is an inverse relationship between education and obesity observed especially for women [36]. Low education level is one of many socioeconomic factors that have been shown to be strong predictors for the risk of obesity [36, 37].

Persons who inject drugs (PWID) comprise 54.3% of our drug user cohort, all of whom have a history of opioid addiction. Previous studies have associated PWID using opioids with being underweight [34, 38, 39]. One plausible explanation may be that opioids may take precedence over seeking nutritional food, leading to long-term poor dietary patterns and nutritional deficiency [34, 38]. The combination of being a PWID and underweight poses a significant health risk. Nutritional deficiencies and malnourishment increase susceptibility to infections. Particularly with PWID, the risk and management of HIV is of great concern [34, 38, 40].

In drug cohort studies during the 1980s, the opioid epidemic was primarily manifested among PWID. However, the opioid epidemic has evolved, perhaps explaining the increase in non-PWID seen in our current cohort. Addiction to post-surgical pain medications has emerged as a common introduction to OUDs. Purity of street opioids has increased, minimizing the extent to which tolerance builds, and costs have plummeted. The introduction of fentanyl to the illegal drug markets has provided many with extreme highs and increasing mortality. In line with the changing demographics of the opioid epidemic, the younger group, who are more likely to have non-PWID patterns of use, showed increased tendency to be overweight (SPR=0.61, p=0.043) or obese (SPR=1.68, p=0.005) than the New Jersey population from NJBRFS. We found the older group (subjects > 50 years old), who tended to use more injection opioids than the younger group (subjects ≤ 50 years old). Older subjects were also more likely to be underweight than rates calculated from NJBRFS and NHIS data (SPR=4.03, p=0.037 and SPR=4.89, p=0.02 respectively). In our data persons who did not inject drugs (non-PWID), which are 45.7% of our cohort, were more likely to be overweight compared to general population calculated from NJBRFS data (SPR=0.58, p=0.027). This is a surprising result, since previous studies have not found a significant relationship between non-PWID and BMI [39].

Our study found that obese subjects were less likely to be regular cannabis users than subjects of normal weight (p=0.011). Cannabis is thought to be an appetite stimulant in low-weight individuals; a phenomenon colloquially termed as “the munchies” [41]. Overeating could be in competition with cannabis in brain reward sites [42]. Our finding suggests that the effect of regular cannabis use may not be a primary contributing factor to the increased obesity prevalence in this cohort; instead, the increased obesity prevalence observed in this cohort is likely multifactorial. Several studies have reported alcohol consumption to be associated with an increased BMI, however in our study we found obese individuals were less likely to be heavy drinkers (p=0.030) [38, 43]. Barry *et al.* found BMI is positively associated with a lifetime risk for alcohol abuse in men and inversely associated with the risk of drinking in the last year for women [43]. Patterns of drinking such as frequency and quantity (ex. binge drinking) must be key considerations in epidemiological research studying alcohol and BMI and illicit drug use [43].

## Limitations

Our paper describes significant epidemiological trends, that have crucial clinical applications. Since this is a prevalence study, we did not collect data regarding environmental factors such as physical activity, nutrition, and dietary habits, all of which are implicated in the etiology of obesity. Future studies should examine the causal relationships between weight gain and methadone doses as patterns differ by race/ethnicity, gender, and age.

Since we used medical records from the MAT facilities, we were unable to verify the consistency of weight and height measurements (i.e., time of day, shoes on or off), which could lead to some degree of error in the BMI calculations. Further, for this analysis we only used the BMI data we had closest to the date of interview, meaning that trends were not assessed. In future surveys, patients should be asked to recall their weight at different points in their lives. Having two points of BMI measurement, one at the time of enrollment and one several years later, would allow documentation of longitudinal changes in BMI trends. Also, BMI cannot account for differences in body makeup, so a fat percentage should also be calculated for each patient.

It is possible that there is some bias in the sampling of the survey data used. For NHIS in particular, 90% of selected participants chose to respond, but the 10% who chose not to, could not be replaced; therefore, their demographics may be underrepresented. It is impossible for us to determine in which direction this would cause the data to be skewed.

## Conclusion

We found varying BMI trends by race/ethnicity, gender, and age in patients with OUD on methadone enrolled in MAT programmes. The prevalence of obesity in females was much higher than in the US population. Also, subjects less than 50 years old were significantly more overweight and obese compared to New Jersey. Surprisingly, persons who did not inject drugs were more overweight. Prevalence of underweight was significantly higher among Black non-Hispanic minorities, males, older subjects aged 66-85, and persons who inject drugs. The BMI variation by race/ethnicity, gender, and age suggests a need for tailoring screening and prevention strategies. MAT clinics and primary care providers must identify vulnerable OUD patients and provide them with education on risk mitigation and implement interventions to improve health outcomes.

## Figures and Tables

**Table 1:** Demographics of Drug User Cohort (N=151).

Category	N (%)	Mean Methadone Dose (mg/day)
Gender		
Female	89 (58.9)	83.1^a^
Male	62 (41.1)	84.4^a^
Race/Ethnicity		
Hispanic (H)	34 (22.5)	90.5
Black non-Hispanic (BNH)	59 (39.1)	77.6^a^
White non-Hispanic (WNH)	55 (36.4)	85.9^a^
Asian non-Hispanic	1 (0.7)	5.0
Other non-Hispanic	2 (1.3)	94.0
Age Ranges		
21-35	23 (15.2)	80.5^a^
36-40	18 (11.9)	84.7^a^
41-45	12 (7.9)	98.3
46-50	21 (13.9)	95.1
51-55	26 (17.2)	73.1^a^
56-60	26 (17.2)	76.1
61-65	16 (10.6)	88.0^a^
66-85	9 (6.0)	73.2^a^
BMI		
Underweight	7 (4.6)	**56.7**^a^ *
Normal Weight	40 (26.5)	81.9^a^
Overweight	40 (26.5)	87.7^a^
Obese	64 (42.4)	91.1^a^
		
Diabetes	26 (17.2)	82.0
No Diabetes	125 (82.8)	84.0^a^
		
Persons Who Inject Drugs (PWID)	82 (54.3)	84.2^a^
Persons Who Did Not Inject Drugs (NON-PWID)	69 (45.7)	83.0^a^
		
Education		
Elementary School [K-5th grade]	1 (0.7)	140.0
Middle School [6-8th grade]	7 (4.6)	95.0
Some High School [9-12th grade]	47 (31.1)	85.2^a^
High School Graduate	40 (26.5)	85.7^a^
GED or equivalent	11 (7.3)	83.2
Some College (No Degree)	26 (17.2)	91.6^a^
Associate Degree or Certificate (Occupational, Technical, or Vocational Programme)	4 (2.6)	80.0
Associate Degree (Academic Programme)	3 (2.0)	123.3
Bachelor’s degree (example: BA, AB, BS, BBA)	8 (5.3)	69.9
Master’s degree (example: MA, MS, MEng, MEd, MBA, MPH)	3 (2.0)	63.3
Professional School Degree (ex: MD, DDS, DVM, JD)	1 (0.7)	70.0

Note: 5 subjects reported that methadone was not a part of their treatment regimen, therefore for mean methadone calculations n=146.

Demographics for the 5 subjects not on methadone who are excluded from the above table:

Subject 1 - WNH female, non-diabetic, PWID, 21 years-old, normal weight, high school graduate.

Subject 2 - WNH female, non-diabetic, non-PWID, 54 years-old, normal weight, some high school [9-12^th^ grade].

Subject 3 - BNH female, non-diabetic, non-PWID, 36 years-old, obese, high school graduate.

Subject 4 - BNH male, non-diabetic, PWID, 61 years-old, overweight, some college (no degree).

Subject 5 - BNH female, non-diabetic, PWID, 66 years-old, underweight, some high school [9-12^th^ grade].

a : One or more subjects in this category are excluded in this mean, as they did not use methadone.

* : In a two-sample independent t-test of equal variance with normal weight as a control, two-tailed p <0.1.

**Table 2:** BMI Comparison: Drug User Cohort to NJBRFS and NHIS data. (N = 151).

	BMI Classification											
	Underweight			Normal Weight			Overweight			Obese		
Category	Cohort	NJBRFS	NHIS	Cohort	NJBRFS	NHIS	Cohort	NJBRFS	NHIS	Cohort	NJBRFS	NHIS
	Obs (% of 151)	Obs/Exp	Obs/Exp	Obs (% of 151)	Obs/Exp	Obs/Exp	Obs (% of 151	Obs/Exp	Obs/Exp	Obs (% of 151)	Obs/Exp	Obs/Exp
**Overall**	7 (4.6)	**3.52** **	**3.98** **	40 (26.5)	0.93	1.00	40 (26.5)	**0.70** *	0.79	64 (42.4)	**1.30** *	1.01
**Hispanic**	1 (0.7)	2.58	5.52	7 (4.6)	0.84	0.91	9 (6.0)	0.61	0.66	17 (11.3)	1.62	1.37
**Black NH**	4 (2.6)	**6.71** **	**7.36** **	14 (9.3)	1.20	1.17	14 (9.3)	0.63	0.73	27 (17.9)	1.11	0.99
**White NH**	2 (1.3)	2.34	2.21	19 (12.6)	0.87	1.00	15 (9.9)	0.80	0.87	19 (12.6)	1.40	1.06
**Male**	3 (2.0)	**7.44** **	**7.69** *	16 (10.6)	1.22	1.13	22 (14.6)	0.76	0.84	21 (13.9)	1.08	0.99
**Female**	4 (2.65)	2.52	2.92	24 (15.9)	0.81	0.92	18 (11.9)	0.65	0.73	43 (28.5)	**1.44** *	1.16
**21-35**	1 (0.7)	1.83	1.91	9 (6.0)	0.84	0.95	4 (2.6)	0.59	0.63	9 (6.0)	1.81	1.35
**36-40**	1 (0.7)	5.26	6.65	4 (2.6)	0.80	0.85	5 (3.3)	0.66	0.72	8 (5.3)	1.54	1.28
**41-45**	1 (0.7)	9.26	7.78	4 (2.6)	1.04	1.18	2 (1.3)	0.46	0.50	5 (3.3)	1.36	1.12
**46-50**	N.C.	N.C.	N.C.	2 (1.3)	0.40	0.47	5 (3.3)	0.64	0.70	14 (9.3)	1.76	1.48
**51-55**	1 (0.7)	3.57	5.20	8 (5.3)	1.25	1.37	6 (4.0)	0.62	0.70	11 (7.3)	1.14	0.97
**56-60**	1 (0.7)	2.53	4.06	7 (4.6)	1.16	1.23	7 (4.6)	0.70	0.80	11 (7.3)	1.16	0.97
**61-65**	N.C.	N.C.	N.C.	4 (2.6)	1.01	0.92	7 (4.6)	1.04	1.24	5 (3.3)	0.99	0.87
**66-85**	N.C.	N.C.	**13.91** *	2 (1.3)	1.03	0.83	N.C.	N.C.	1.16	1 (0.7)	0.31	0.33
**Younger Group (Subjects ≤50)**	3 (2.0)	3.01	3.18	18 (11.9)	0.74	0.87	16 (10.6)	**0.61** *	0.66	36 (23.8)	**1.68** **	1.34
**Older Group (Subjects >50)**	4 (2.6)	**4.03** **	**4.89** *	22 (14.6)	1.18	1.15	24 (15.9)	0.79	0.91	28 (18.5)	1.01	0.89
**PWID**	5 (3.3)	**4.15** *	**4.37** *	23 (15.2)	0.75	0.94	25 (16.6)	0.81	0.91	29 (19.2)	1.22	1.01
**Non-PWID**	17 (11.2)	2.55	3.27	15 (9.9)	1.02	1.09	35 (23.2)	**0.58** *	0.65	4 (2.6)	1.38	1.19

Note: Standardized Prevalence Ratios were calculated to examine differences between drug user cohort, NJBRFS, and NHIS data. Boldface indicates statistical significance in two-tailed Fisher exact test (* p < 0.05 and ** p < 0.01).

PWID: Persons who inject drugs.

N.C.: Non-Calculable due to absence of data in the reference database, which excluded data when those databases had small numbers of persons in the cells; NH: Non-Hispanic.

**Table 3:** The Association of BMI with Regular Cannabis Use and Alcohol Use.

	Regular Cannabis Use			Alcohol Use			
BMI Category	Never Regular Cannabis Use (N=73)	Regular Cannabis Use (N=78)	RR for weight category	Abstained (N=42)	Light/ Moderate (N=42)	Heavy (N=67)	RR for weight category (heavy alcohol use vs. abstained)
**Underweight**	3 (2.0)	4 (2.6)	0.76	3 (2.0)	2 (1.3)	2 (1.3)	0.27
**Normal Weight**	14 (9.3)	26 (17.2)	----	7 (4.6)	10 (6.6)	23 (15.2)	----
**Overweight**	16 (10.6)	24 (15.9)	0.90	10 (6.6)	9 (6.0)	21 (13.9)	0.81
**Obese**	40 (26.5)	24 (15.9)	0.65*	22 (14.6)	21 (13.9)	21 (13.9)	0.63*

Note: Risk ratio was calculated in comparison to “normal weight” as the reference group.

Boldface indicates statistical significance through two-tailed Fisher exact test, p <0.05.

Regular Cannabis Use RR compares risks of BMI categories Underweight, Overweight and Obese to Normal Weight between regular cannabis users and never cannabis users.

Alcohol Use RR compares risks of BMI categories Underweight, Overweight, and Obese to Normal Weight between heavy alcohol users and abstainers.

**Table 4:** BMI comparison: Drug User Cohort to NJBRFS Data. (N=151).

BMI Classifications																				
	Underweight					Normal Weight					Overweight					Obese				
		
	Cohort		NJBRFS			Cohort		NJBRFS			Cohort		NJBRFS			Cohort		NJBRFS		
																			
	Obs (% of 151)	Exp	Obs/Exp	p-value	95% CI	Obs (% of 151)	Exp	Obs/Exp	p-value	95% CI	Obs (% of 151)	Exp	Obs/Exp	p-value	95% CI	Obs (% of 151)	Exp	Obs/Exp	p-value	95% CI
**Overall**	7 (4.6)	1.99	3.52	**0.009** **	1.41-7.25	40 (26.5)	42.9	0.93	0.731	0.67-1.27	40 (26.5)	56.8	0.70	**0.024** *	0.50-0.96	64 (42.4)	49.3	1.30	**0.049** *	1.00-1.66
**Hispanic**	1 (0.7)	0.39	2.58	0.642	0.07-14.40	7 (4.6)	8.30	0.84	0.824	0.34-1.74	9 (6.0)	14.7	0.61	0.158	0.28-1.16	17 (11.3)	10.5	1.62	0.081	0.94-2.59
**Black NH**	4 (2.6)	0.60	6.71	**0.007** **	1.83-17.18	14 (9.3)	11.7	1.20	0.578	0.65-2.01	14 (9.3)	22.3	0.63	0.084	0.34-1.05	27 (17.9)	24.4	1.11	0.649	0.73-1.61
**White NH**	2 (1.3)	0.86	2.34	0.422	0.28-8.45	19 (12.6)	21.8	0.87	0.642	0.52-1.36	15 (9.9)	18.8	0.80	0.463	0.45-1.32	19 (12.6)	13.6	1.40	0.194	0.84-2.18
**Male**	3 (2.0)	0.40	7.44	**0.016** *	1.54-21.76	16 (10.6)	13.1	1.22	0.496	0.70-1.98	22 (14.6)	29.0	0.76	0.224	0.48-1.15	21 (13.9)	19.5	1.08	0.789	0.67-1.65
**Female**	4 (2.6)	1.59	2.52	0.154	0.69-6.45	24 (15.9)	29.8	0.81	0.334	0.52-1.20	18 (11.9)	27.9	0.65	0.063	0.38-1.02	43 (28.5)	29.8	1.44	**0.026** *	1.05-1.95
**21-35**	1 (0.7)	0.55	1.83	0.844	0.05-10.17	9 (6.0)	10.7	0.84	0.750	0.38-1.60	4 (2.6)	6.79	0.59	0.386	0.16-1.51	9 (6.0)	4.97	1.81	0.132	0.83-3.44
**36-40**	1 (0.7)	0.19	5.26	0.346	0.13-29.33	4 (2.6)	5.02	0.80	0.875	0.22-2.04	5 (3.3)	7.60	0.66	0.461	0.21-1.54	8 (5.3)	5.18	1.54	0.306	0.67-3.04
**41-45**	1 (0.7)	0.11	9.26	0.205	0.23-51.59	4 (2.6)	3.83	1.04	1.000	0.28-2.67	2 (1.3)	4.38	0.46	0.376	0.06-1.65	5 (3.3)	3.68	1.36	0.619	0.44-3.17
**46-50**	N.C.	N.C.	N.C.	N.C.	N.C.	2 (1.3)	4.99	0.40	0.250	0.05-1.45	5 (3.3)	7.86	0.64	0.409	0.21-1.49	14 (9.3)	7.96	1.76	0.066	0.96-2.95
**51-55**	1 (0.7)	0.28	3.57	0.488	0.09-19.90	8 (5.3)	6.40	1.25	0.626	0.54-2.46	6 (4.0)	9.66	0.62	0.306	0.23-1.35	11 (7.3)	9.64	1.14	0.743	0.57-2.04
**56-60**	1 (0.7)	0.40	2.53	0.653	0.06-14.11	7 (4.6)	6.05	1.16	0.803	0.47-2.38	7 (4.6)	10.1	0.70	0.429	0.28-1.43	11 (7.3)	9.49	1.16	0.706	0.58-2.08
**61-65**	N.C.	N.C.	N.C.	N.C.	N.C.	4 (2.6)	3.97	1.01	1.000	0.27-2.58	7 (4.6)	6.74	1.04	1.000	0.42-2.14	5 (3.3)	5.08	0.99	0.794	0.32-2.30
**66-85**	N.C.	N.C.	N.C.	N.C.	N.C.	2 (1.3)	1.95	1.03	1.000	0.12-3.71	N.C.	N.C.	N.C.	N.C.	N.C.	1 (0.7)	3.26	0.31	0.327	0.01-1.71
**Younger Group (Subjects ≤50)**	3 (2.0)	1.00	3.01	0.160	0.62-8.79	18 (11.9)	24.2	0.74	0.239	0.44-1.18	16 (10.6)	26.3	0.61	**0.043** *	0.35-0.99	36 (23.8)	21.4	1.68	**0.005** **	1.18-2.32
**Older Group (Subjects >50)**	4 (2.6)	0.99	4.03	**0.037** *	1.10-10.31	22 (14.6)	18.7	1.18	0.501	0.74-1.78	24 (15.9)	30.5	0.79	0.274	0.50-1.17	28 (18.5)	27.8	1.01	1.000	0.67-1.46
**PWID**	5 (3.3)	1.21	4.15	**0.016** *	1.35-9.68	23 (15.2)	30.7	0.75	0.183	0.47-1.12	25 (16.6)	30.7	0.81	0.345	0.53-1.20	29 (19.2)	23.8	1.22	0.334	0.82-1.75
**Non-PWID**	2 (1.3)	0.79	2.55	0.372	0.31-9.20	17 (11.3)	16.7	1.02	1.000	0.59-1.63	15 (9.9)	26.1	0.58	**0.027** *	0.32-0.95	35 (23.2)	25.4	1.38	0.083	0.96-1.91

Note: Standardized Prevalence Ratios were calculated to examine differences between drug user cohort and NJBRFS data. Boldface indicates statistical significance in two-tailed Fisher exact test (* p < 0.05 and ** p < 0.01).

PWID: Persons Who Inject Drugs; N.C.: Non-Calculable due to insufficient data; NH: Non-Hispanic.

**Table 5:** BMI comparison: Drug User Cohort to NHIS Data. (N=151).

	BMI Classification																			
	Underweight					Normal Weight					Overweight					Obese				
	Cohort	NHIS				Cohort	NHIS				Cohort	NHIS				Cohort	NHIS			
	Obs (% of 151)	Exp	Obs/Exp	p-value	95% CI	Obs (% of 151)	Exp	Obs/Exp	p-value	95% CI	Obs (% of 151)	Exp	Obs/Exp	p-value	95% CI	Obs (% of 151)	Exp	Obs/Exp	p-value	95% CI
**Overall**	7 (4.6)	1.76	3.98	**0.005** **	1.60-8.19	40 (26.5)	40.2	1.00	0.936	0.71-1.36	40 (26.5)	50.8	0.79	0.141	0.56-1.07	64 (42.4)	58.3	1.01	0.488	0.85-1.40
**Hispanic**	1 (0.7)	0.18	5.52	0.332	0.14-30.73	7 (4.6)	7.66	0.91	0.997	0.37-1.88	9 (6.0)	13.7	0.66	0.246	0.30-1.24	17 (11.3)	12.4	1.37	0.253	0.80-2.19
**Black NH**	4 (2.6)	0.54	7.36	**0.005** **	2.00-18.83	14 (9.3)	12.0	1.17	0.633	0.64-1.96	14 (9.3)	19.1	0.73	0.287	0.40-1.23	27 (17.9)	27.4	0.99	0.952	0.65-1.44
**White NH**	2 (1.3)	0.91	2.21	0.459	0.27-7.98	19 (12.6)	19.0	1.00	0.883	0.60-1.56	15 (9.9)	17.2	0.87	0.708	0.49-1.44	19 (12.6)	17.9	1.06	0.853	0.64-1.66
**Male**	3 (2.0)	0.39	7.69	**0.015** *	1.59-22.48	16 (10.6)	14.1	1.13	0.685	0.65-1.84	22 (14.6)	26.2	0.84	0.477	0.53-1.27	21 (13.9)	21.3	0.99	0.931	0.61-1.51
**Female**	4 (2.6)	1.37	2.92	0.101	0.80-7.48	24 (15.9)	26.0	0.92	0.786	0.59-1.37	18 (11.9)	24.6	0.73	0.213	0.43-1.16	43 (28.5)	37.0	1.16	0.365	0.84-1.57
**21-35**	1 (0.7)	0.52	1.91	0.814	0.05-10.66	9 (6.0)	9.49	0.95	0.955	0.43-1.80	4 (2.6)	6.34	0.63	0.484	0.17-1.62	9 (6.0)	6.64	1.35	0.452	0.62-2.57
**36-40**	1 (0.7)	0.15	6.65	0.279	0.17-37.07	4 (2.6)	4.69	0.85	1.000	0.23-2.19	5 (3.3)	6.90	0.72	0.627	0.24-1.69	8 (5.3)	6.26	1.28	0.586	0.55-2.52
**41-45**	1 (0.7)	0.13	7.78	0.241	0.20-43.33	4 (2.6)	3.39	1.18	0.878	0.32-3.02	2 (1.3)	4.01	0.50	0.472	0.06-1.80	5 (3.3)	4.47	1.12	0.924	0.36-2.61
**46-50**	N.C.	N.C.	N.C.	N.C.	N.C.	2 (1.3)	4.30	0.47	0.396	0.06-1.68	5 (3.3)	7.12	0.70	0.571	0.23-1.64	14 (9.3)	9.44	1.48	0.197	0.81-2.49
**51-55**	1 (0.7)	0.19	5.20	0.350	0.13-28.97	8 (5.3)	5.85	1.37	0.470	0.59-2.70	6 (4.0)	8.56	0.70	0.499	0.26-1.53	11 (7.3)	11.4	0.97	0.936	0.48-1.73
**56-60**	1 (0.7)	0.25	4.06	0.436	0.10-22.64	7 (4.6)	5.72	1.23	0.696	0.49-2.52	7 (4.6)	8.74	0.80	0.710	0.32-1.65	11 (7.3)	11.3	0.97	0.912	0.49-1.74
**61-65**	N.C.	N.C.	N.C.	N.C.	N.C.	4 (2.6)	4.34	0.92	0.874	0.25-2.36	7 (4.6)	5.66	1.24	0.679	0.50-2.55	5 (3.3)	5.76	0.87	1.000	0.28-2.02
**66-85**	2 (1.3)	0.14	13.91	**0.019** *	1.68-50.24	2 (1.3)	2.40	0.83	0.860	0.10-3.01	4 (2.6)	3.44	1.16	0.902	0.32-2.98	1 (0.7)	3.01	0.33	0.394	0.01-1.85
**Younger Group (Subjects ≤50)**	3 (2.0)	0.94	3.18	0.140	0.66-9.30	19 (12.6)	21.9	0.87	0.633	0.52-1.36	16 (10.6)	24.4	0.66	0.097	0.38-1.07	36 (23.8)	26.8	1.34	0.104	0.94-1.86
**Older Group (Subjects >50)**	4 (2.6)	0.82	4.89	**0.020** *	1.33-12.53	21 (13.9)	18.3	1.15	0.587	0.71-1.75	24 (15.9)	26.4113	0.91	0.731	0.58-1.35	28 (18.5)	31.5	0.89	0.611	0.59-1.29
**PWID**	5 (3.3)	1.15	4.37	**0.013** *	1.42-10.19	23 (15.2)	24.5	0.94	0.864	0.60-1.41	25 (16.6)	27.5	0.91	0.719	0.59-1.34	29 (19.2)	28.8	1.01	1.000	0.67-1.45
**Non-PWID**	2 (1.3)	0.61	3.27	0.252	0.40-11.80	17 (11.3)	15.7	1.09	0.800	0.63-1.74	15 (9.9)	23.3	0.65	0.094	0.36-1.06	35 (23.2)	29.5	1.19	0.353	0.83-1.65

Note: Standardized Prevalence Ratios were calculated to examine differences between drug user cohort and NHIS data. Boldface indicates statistical significance in two-tailed Fisher exact test (* p < 0.05 and ** p < 0.01).

PWID: Persons Who Inject Drugs; N.C.: Non-Calculable due to insufficient data; NH: Non-Hispanic.

**Table 6:** The Association of BMI with Regular Cannabis Use and Alcohol Use.

	Regular Cannabis Use					Alcohol Use					
BMI Category	Regular Cannabis Use (N=78)	Never Regular Cannabis Use (N=73)	RR	p-value	95% CI	Abstained (N=42)	Light/ Moderate (N=42)	Heavy (N=67)	RR	p-value	95% CI
**Underweight**	4 (2.6)	3 (2.0)	0.76	0.998	0.19-2.98	3 (2.0)	2 (1.3)	2 (1.3)	0.27	0.256	0.05-1.36
**Normal Weight**	26 (17.2)	14 (9.3)	----			7 (4.6)	10 (6.6)	23 (15.2)	----		
**Overweight**	24 (15.9)	16 (10.6)	0.90	0.818	0.58-1.40	10 (6.6)	9 (6.0)	21 (13.9)	0.81	0.624	0.49-1.34
**Obese**	24 (15.9)	40 (26.5)	0.65	0.011*	0.47-0.90	22 (14.6)	21 (13.9)	21 (13.9)	0.63	0.030*	0.43-0.91

Note: Risk ratio was calculated. Boldface indicates statistical significance through two-tailed Fisher exact test, p <0.05. Confidence interval calculated as Taylor Series.

Regular Cannabis Use RR compares BMI categories Underweight, Overweight, and Obese to Normal Weight.

Alcohol Use RR compares BMI categories Underweight, Overweight, and Obese to Normal Weight.

## References

[1] Manchikanti L, Singh A (2008) Therapeutic opioids: a ten-year perspective on the complexities and complications of the escalating use, abuse, and nonmedical use of opioids. Pain Physician 11: S63–S88. 18443641

[2] CDC (2022) Adult Obesity Facts. Centers for Disease Control and Prevention. Accessed: 7-31-2020

[3] Ball JC, Ross A (1991) The Effectiveness of Methadone Maintenance Treatment. 1st ed. Springer-Verlag.

[4] Fenn JM, Laurent JS, Sigmon SC (2015) Increases in body mass index following initiation of methadone treatment. J Subst Abuse Treat 51: 59–63. 25441923 10.1016/j.jsat.2014.10.007 PMC4346498

[5] Cooper OB, Brown TT, Dobs AS (2003) Opiate drug use: a potential contributor to the endocrine and metabolic complications in human immunodeficiency virus disease. Clin Infect Dis 37: S132–S136. 12942387 10.1086/375879

[6] Fareed A, Byrd Sellers J, Vayalapalli S, Drexler K, Phillips L (2013) Predictors of Diabetes Mellitus and Abnormal Blood Glucose in Patients Receiving Opioid Maintenance Treatment. Am J Addict 22: 411–416. 23795882 10.1111/j.1521-0391.2013.12043.x

[7] Mysels DJ, Sullivan MA (2010) The relationship between opioid and sugar intake: review of evidence and clinical applications. J Opioid Manag 6: 445–452. 21269006 10.5055/jom.2010.0043 PMC3109725

[8] Gambera SE, Clarke JA (1976) Comments on dietary intake of drug-dependent persons. J Am Diet Assoc 68: 155–157. 1245716

[9] Nolan LJ (2013) Shared Urges? The Links Between Drugs of Abuse, Eating, and Body Weight. Current Obesity Reports 2: 150–156.

[10] Volkow ND, Wang GJ, Baler RD (2011) Reward, dopamine and the control of food intake: implications for obesity. Trends Cogn Sci 15: 37–46. 21109477 10.1016/j.tics.2010.11.001 PMC3124340

[11] Volkow ND, Wise RA (2005) How can drug addiction help us understand obesity? Nat Neurosci 8: 555–560. 15856062 10.1038/nn1452

[12] Volkow ND, Baler RD (2015) NOW vs LATER brain circuits: implications for obesity and addiction. Trends Neurosci 38: 345–352. 25959611 10.1016/j.tins.2015.04.002

[13] Volkow ND, Wise RA, Baler R (2017) The dopamine motive system: implications for drug and food addiction. Nat Rev Neurosci 18: 741–752. 29142296 10.1038/nrn.2017.130

[14] Vallecillo G, Robles MJ, Torrens M, Samos P, Roquer A (2018) Metabolic syndrome among individuals with heroin use disorders on methadone therapy: Prevalence, characteristics, and related factors. Subst Abus 39: 46–51. 28771091 10.1080/08897077.2017.1363122

[15] Peles E, Schreiber S, Sason A, Adelson M (2016) Risk factors for weight gain during methadone maintenance treatment. Subst Abus 37: 613–618. 27093441 10.1080/08897077.2016.1179705

[16] Dietz W, Santos Burgoa C (2020) Obesity and its Implications for COVID-19 Mortality. Obesity 28: 1005. 32237206 10.1002/oby.22818

[17] Michalakis K, Panagiotou G, Ilias I, Pazaitou Panayiotou K (2021) Obesity and COVID-19: A jigsaw puzzle with still missing pieces. Clin Obes 11: e12420. 33073512 10.1111/cob.12420 PMC7645965

[18] Kassir R (2020) Risk of COVID-19 for patients with obesity. Obes Rev 21: e13034. 32281287 10.1111/obr.13034 PMC7235532

[19] National Institute on Drug Abuse (2022) COVID-19 & Substance Use. National Institute on Drug Abuse; Accessed: 6-29-2022.

[20] Aldington S, Williams M, Nowitz M, Weatherall M, Pritchard A (2007) Effects of cannabis on pulmonary structure, function and symptoms. Thorax 62: 1058–1063. 17666437 10.1136/thx.2006.077081 PMC2094297

[21] Plein LM, Rittner HL (2018) Opioids and the immune system - friend or foe. Br J Pharmacol 175: 2717–2725. 28213891 10.1111/bph.13750 PMC6016673

[22] Wang QQ, Kaelber DC, Xu R, Volkow ND (2021) COVID-19 risk and outcomes in patients with substance use disorders: analyses from electronic health records in the United States. Mol Psychiatry 26: 30–39. 32929211 10.1038/s41380-020-00880-7 PMC7488216

[23] Muñoz Price LS, Nattinger AB, Rivera F, Hanson R, Gmehlin CG (2020) Racial Disparities in Incidence and Outcomes Among Patients With COVID-19. JAMA Netw Open 3: e2021892. 32975575 10.1001/jamanetworkopen.2020.21892 PMC7519420

[24] The Lancet (2020) The plight of essential workers during the COVID-19 pandemic. Lancet 395: 1587. 32446399 10.1016/S0140-6736(20)31200-9 PMC7241973

[25] Knox KR, Weiss SH (2001) Comparison of Cancer Incidence in HIV+ and HIV− Injection Drug Users: 15-Year Follow Up of a Cohort Study [abstract]. New Jersey Medical School Summer Student Research Abstracts 64–66, Newark, New Jersey, USA.

[26] New Jersey Department of Health (2020) New Jersey State Health Assessment Data. Accessed: 4-18-2020.

[27] National Center for Health Statistics (2020) National Center for Health Statistics. Data File Documentation, National Health Interview Survey, 2011-2017. Hyattsville, Maryland: CDC.

[28] Breslow NE, Day NE (1987) Statistical Methods in Cancer Research: Volume II - The Design and Analysis of Cohort Studies. 82 ed. Lyon, France: International Agency for Research on Cancer; IARC Scientific Publications. 3329634

[29] OpenEpi (2013) Open Source Epidemiologic Statistics for Public Health, Version 3.01. Atlanta, GA.

[30] Soe MM, Sullivan KM (2005) Two-sample Independent *t* Test. In: Dean AG, Sullivan KM, Soe MM, eds., Accessed: 6-29-2022. http://openepi.com/PDFDocs/t_testMeanDoc.pdf

[31] U.S. Department of Health and Human Services Office of Minority Health. Obesity and African Americans. 2020. Accessed: 7-31-2020.

[32] Hendley Y, Zhao L, Coverson DL, Dzietham RD, Morris A (2011) Differences in weight perception among blacks and whites. J Womens Health 20: 1805–1811. 10.1089/jwh.2010.2262 PMC3236990 21988528

[33] Levi J, Segal L, St. Laurent R, Rayburn J (2014) The State of Obesity: Better Policies for a Healthier America. Trust for America's Health. Accessed 8-14-2020.

[34] Nazrul Islam SK, Hossain KJ, Ahmed A, Ahsan M (2002) Nutritional status of drug addicts undergoing detoxification: prevalence of malnutrition and influence of illicit drugs and lifestyle. Br J Nutr 88: 507–513. 12425731 10.1079/BJN2002702

[35] Vera Villarroel P, Piqueras JA, Kuhne W, Cuijpers P, van Straten A (2014) Differences between men and women in self-reported body mass index and its relation to drug use. Subst Abuse Treat Prev Policy 9:1. 24383608 10.1186/1747-597X-9-1 PMC3880835

[36] Cohen AK, Rai M, Rehkopf DH, Abrams B (2013) Educational attainment and obesity: a systematic review. Obes Rev 14: 989–1005. 23889851 10.1111/obr.12062 PMC3902051

[37] Arroyo Johnson C, Mincey KD (2016) Obesity epidemiology trends by race/ethnicity, gender, and education: National Health Interview Survey, 1997–2012. Gastro Clin North Am 45: 571–579. 10.1016/j.gtc.2016.07.012 PMC5599163 27837773

[38] McIlwraith F, Betts KS, Jenkinson R, Hickey S, Burns L (2014) Is low BMI associated with specific drug use among injecting drug users? Subst Use Misuse 49: 374–382. 24102254 10.3109/10826084.2013.841246

[39] Chatterjee S, Tempalski B, Pouget ER, Cooper HLF, Cleland CM (2011) Changes in the prevalence of injection drug use among adolescents and young adults in large U.S. metropolitan areas. AIDS Behav 15: 1570–1578. 21739288 10.1007/s10461-011-9992-0 PMC3299409

[40] Quach LA, Wanke CA, Schmid CH, Gorbach SL, Mwamburi DM (2008) Drug use and other risk factors related to lower body mass index among HIV-infected individuals. Drug Alcohol Depend 95: 30–36. 18243579 10.1016/j.drugalcdep.2007.12.004 PMC3837518

[41] Sansone RA, Sansone LA (2014) Marijuana and body weight. Innov Clin Neurosci 11: 50–54. 25337447 PMC4204468

[42] Warren M, Frost Pineda K, Gold M (2005) Body mass index and marijuana use. J Addict Dis 24: 95–100. 16186086 10.1300/J069v24n03_08

[43] Barry D, Petry NM (2009) Associations between body mass index and substance use disorders differ by gender: results from the National Epidemiologic Survey on Alcohol and Related Conditions. Addict Behav 34: 51–60. 18819756 10.1016/j.addbeh.2008.08.008 PMC2645714

